# Contribution of GlyR α3 Subunits to the Sensitivity and Effect of Ethanol in the Nucleus Accumbens

**DOI:** 10.3389/fnmol.2021.756607

**Published:** 2021-10-22

**Authors:** Loreto S. San Martin, Lorena Armijo-Weingart, Anibal Araya, Gonzalo E. Yévenes, Robert J. Harvey, Luis G. Aguayo

**Affiliations:** ^1^Department of Physiology, Programa de Neurociencia, Psiquiatria y Salud Mental, Universidad de Concepción, Concepción, Chile; ^2^School of Health and Behavioural Sciences, University of the Sunshine Coast, Sunshine Coast, QLD, Australia; ^3^Sunshine Coast Health Institute, Birtinya, QLD, Australia

**Keywords:** glycine receptor (GlyR), ethanol, nucleus accumbens, alcohol use disorder (AUD), reward system

## Abstract

The glycine receptor (GlyR), a ligand-gated ion channel, is critical for inhibitory neurotransmission in brainstem, spinal cord, and in supraspinal regions. Recent data from several laboratories have shown that GlyRs are expressed in the brain reward circuitry and that α1 and α2 are the principal subunits expressed in the nucleus accumbens (nAc). In the present study, we studied the sensitivity to ethanol of homomeric and heteromeric α3 GlyR subunits in HEK293 cells and dissociated neurons from the nAc. Finally, we explored ethanol-related behaviors in a *Glra3* knockout mouse (*Glra3*^–/–^). Studies in HEK293 cells showed that while homomeric α3 GlyR subunits were insensitive to ethanol, heteromeric α3β GlyR subunits showed higher sensitivity to ethanol. Additionally, using electrophysiological recordings in dissociated accumbal neurons, we found that the glycine current density increased in *Glra3*^–/–^ mice and the GlyRs were less affected by ethanol and picrotoxin. We also examined the effect of ethanol on sedation and drinking behavior in *Glra3*^–/–^ mice and found that the duration in the loss of righting reflex (LORR) was unchanged compared to wild-type (WT) mice. On the other hand, using the drinking in the dark (DID) paradigm, we found that *Glra3*^–/–^ mice have a larger ethanol consumption compared to WT mice, and that this was already high during the first days of exposure to ethanol. Our results support the conclusion that heteromeric α3β, but not homomeric α3, GlyRs are potentiated by ethanol. Also, the increase in GlyR and GABA_*A*_R mediated current densities in accumbal neurons in the KO mice support the presence of compensatory changes to α3 knock out. The increase in ethanol drinking in the *Glra3*^–/–^ mice might be associated to the reduction in β and compensatory changes in other subunits in the receptor arrangement.

## Introduction

The nucleus accumbens (nAc) is a key region in the mesolimbic dopaminergic system and is important in the mediation of goal-directed behaviors, motivational processes, and addiction-related behaviors. The nAc receives dopaminergic input from the ventral tegmental area (VTA) and excitatory input from the prefrontal cortex, hippocampus, and amygdala (Russo and Nestler, 2013). It is well known that pharmacological doses of alcohol (ethanol) activate the mesolimbic dopaminergic reward system, increasing dopamine levels in the nAc (Di Chiara and Imperato, 1988; Molander and Soderpalm, 2005a). Therefore, changes in the excitation-inhibition balance would affect accumbal activity and likely change motivation and addictive behaviors.

The glycine receptor (GlyR) is the main inhibitory receptor in the brainstem and spinal cord (Legendre, 2001). Recent studies have demonstrated the presence of GlyRs in higher brain regions such as the prefrontal cortex (Lu and Ye, 2011; Salling and Harrison, 2014), nAc (Martin and Siggins, 2002; Munoz et al., 2018), and VTA (Ye et al., 2001; Li et al., 2012). Functional GlyRs are pentameric complexes composed of α and β subunits that can assemble to form homomeric (5α) or heteromeric (2α3β) channels. To date, molecular cloning studies have demonstrated four isoforms of the GlyR α subunit (α1-4) and one β subunit (Legendre, 2001; Aguayo et al., 2004; Lynch, 2009). Previous studies have demonstrated that ethanol potentiates the function of GlyRs by a mechanism including the activation of G protein βγ dimers (Yevenes et al., 2008, 2010). However, the behavioral role of brain GlyRs in ethanol-induced effects is only now beginning to emerge. For instance, data from several laboratories have shown that GlyRs are functional in the brain reward system and that α1 and α2 are the predominant subunits expressed in the nAc (Jonsson et al., 2012; Forstera et al., 2017). Our laboratory recently characterized the properties of GlyRs in the nAc (Forstera et al., 2017) and showed that accumbal medium spiny neurons (MSNs) express functional, ethanol-sensitive GlyRs, primarily α1 and α2 subunits. However, the presence of GlyR α3 subunits was not ruled out because mRNA was detected in the nAc (Jonsson et al., 2012; Forstera et al., 2017). More recently, the presence of α3 subunit GlyRs in accumbal MSNs was suggested (McCracken et al., 2017). Unlike the present study, using brain slice and local glycine application, the authors reported that mice with a deletion in *Glra3* (*Glra3*^–/–^ mice) lacked tonic glycinergic currents in the striatum suggesting that α3 subunits play a role in the activation of tonically activated GlyRs. Interestingly, *Glra3*^–/–^ mice showed increased ethanol intake and preference in the 24-h intermittent access protocols (Blednov et al., 2015). However, previous studies using recombinant α3 subunits in HEK293 cells suggested that homomeric α3 GlyRs were insensitive to pharmacologically relevant concentrations of ethanol (Sanchez et al., 2015). On the other hand, the presence of GlyR β subunits was shown in the nAc using immunodetection experiments (Forstera et al., 2017; Munoz et al., 2018; San Martin et al., 2020), thus indicating the presence of heteromeric α3β GlyRs in this region. Therefore, it is possible that ethanol potentiates α3β, but not α3 homomeric complexes, a subunit arrangement that is likely present in the nAc, but this possibility has not yet been examined.

In this study, we first investigated whether the presence of β subunits in the GlyR complex resulted in ethanol sensitivity of α3β GlyRs in HEK293 cells. Second, we studied the differential sensitivity to picrotoxin (PTX) of homomeric and heteromeric α3 GlyR subunits to then test if neurons in the nAc express a heteromeric α3β receptor. Third, we studied the ethanol sensitivity of accumbal neurons from *Glra3*^–/–^ mice. Although neurobiological compensations cannot be ruled out in the *Glra3*^–/–^ mice, especially early during brain development, our data suggest a new role of GlyR α3β complexes in a region important for motivational and addictive behavior.

## Materials and Methods

### Animals

C57BL/6J (WT) and *Glra3* knockout mice aged ∼10 weeks were used in this study. *Glra3* knockout mice were initially generated in the laboratory of Harvey et al. (2004). Breeding pairs were transferred from Switzerland to Chile where they were bred and maintained in a 12h light/dark cycle. *Glra3* knockout mice were backcrossed to C57BL/6J (IMSR Cat# JAX:000664, RRID:IMSR_JAX:000664 and genotyped as described previously (Harvey et al., 2004). All the animals used in this study were generated from crosses between heterozygous males (*Glra3^+/–^*) and heterozygous females (*Glra3^+/–^*). Animal care and experimental procedures were approved by the Institutional Animal Care and Use Committee of the University of Concepción and conducted according to the ethical protocols established by the National Institutes of Health (NIH, Bethesda, MD, United States).

### Experimental Protocol

All the studies were designed to generate groups of equal size and randomly assigned. Operator and data analyses were blinded.

### HEK293 Cell Culture and Transfection

Human embryonic kidney (HEK) 293 cells were cultured using standard methodologies. For glycine-evoked current recordings, HEK293 cells were transfected with GlyR α3L, α3S, and β subunits using an XfectTM Transfection Reagent kit (Clontech, Mountain View, CA, United States). The accession number for the rat GlyR α3L subunit is NM_053724.3. The rat GlyR α3S subunit is identical to this sequence, but lacks a short 45bp cassette exon encoding the sequence TEAFALEKFYRFSDT in the M3-M4 intracellular loop. The accession number for the rat GlyR β subunit is NM_053296.1. Expression of green fluorescent protein (GFP) was used as a marker of positively transfected cells and recordings were made after 18–36 h.

### Preparation of Brain Slices

Wild-type and *Glra3*^–/–^ mice were decapitated as previously described (Forstera et al., 2017). Coronal slices were prepared immediately after excision and placement of the brain in ice-cold cutting solution (in mM: sucrose 194, NaCl 30, KCl 4.5, MgCl_2_ 1, NaHCO_3_ 26, NaH_2_PO_4_ 1.2 and glucose 10, saturated with 95% O_2_/5% CO_2_ and adjusted to pH 7.4), glued to the chilled stage of a vibratome (Leica VT1200S, Leica Biosystems, Germany), and sliced to a thickness of 300 μm. Slices were transferred to an artificial cerebrospinal fluid (aCSF) solution (in mM: NaCl 124, KCl 4.5, MgCl_2_ 1, NaHCO_2_ 26, NaH_2_PO_4_ 1.2, glucose 10 and CaCl_2_ 2, pH 7.4, 315-320 mOsm) saturated with 95% O_2_/5% CO_2_ at 30°C for at least 1 h.

### Enzymatic Dissociation of Accumbal Neurons

For enzymatic dissociation, brain slices that contained the nAc were incubated for 30 min in normal aCSF (saturated with 95% O_2_/5% CO_2_) in the presence of 0.5 mg/mL of pronase (Calbiochem/EDM Bioscience, Darmstadt, Germany) at 37°C. The nAc was dissected from the slices and the tissue was triturated through a series of pipette tips of decreasing diameter size in a 35 mm-culture dish in trituration buffer [in mM: NaCl 20, N-methyl-D-glucamine (NMG) 130, KCl 2.5, MgCl_2_ 1, Hepes 10, glucose 10, adjusted to pH 7.4 and 340 mOsm]. After 20 min, isolated neurons were attached to the bottom of the culture dish and were ready for electrophysiological experiments.

### Electrophysiology

Recordings were made using an Axopatch 200B amplifier (Axon Instrument, Union City, California) at a holding potential of (-60 mV). Currents were displayed and stored on a personal computer using a 1322A Digidata (Axon Instruments) and analyzed with Clampfit 10.1 (Axon Instruments) and MiniAnalysis 6.0 (Synaptosoft, Inc). Patch pipettes with a resistance of 4—6 MΩ were prepared from filament-containing borosilicate micropipettes (World Precision Instruments, Sarasota, FL, USA United States) using a P-87 micropipette puller (Sutter Instrument, Novato, CA, United States) and filled with an internal solution (in mM: KCl 120, MgCl_2_ 4.0, BAPTA 10, Na_2_-GTP 0.5 and Na_2_-ATP 2.0, pH 7.4 and 290–310 mOsm).

Glycine-activated currents were studied in HEK293 cells and dissociated accumbal neurons using whole-cell recordings and an external solution containing (in mM): NaCl 150, KCl 5.4, CaCl_2_ 2.0, MgCl_2_ 1.0, glucose 10 and HEPES 10 (pH 7.4, 315-320 mOsm). We used an array of external tubes (internal diameter, 200 μm) placed within 50 μm of the cell and solutions containing the ligands flowed continuously from the tubes by gravity. The amplitude of the glycine current was measured by using a short pulse (1–2 s) of different concentrations of glycine to perform the concentration-response curve and determine EC_50_ values. For ethanol potentiation, the EC_10_ of glycine was used in the presence or absence of 10–100 mM ethanol. Picrotoxin sensitivity was measured according to a previously described protocol using an EC_30_ of glycine and 20 μM of PTX at a holding potential of −30 mV (Maleeva et al., 2017). A brief pulse of 1 mM glycine was applied at the end of the recording period in every cell to test that the glycine concentration used actually corresponded to EC_10_ or EC_30_. Cells that displayed responses < EC_5_ or > EC_15_ in the case of ethanol sensitivity, or < EC_25_ or > EC_35_ in the case of PTX sensitivity were discarded.

### Western Blots

Tissue homogenates from nAc (50 μg) after lysis treatment (10 mM Tris-HCl pH 7.4, 0.25 M sucrose, 10 mM N-ethylmaleimide (NEM), and protease inhibitor cocktail 1X) were loaded in a 10% sodium dodecyl sulfate-polyacrylamide gel (SDS-PAGE) and placed in an electrophoresis chamber. Subsequently, proteins were blotted onto nitrocellulose membranes, blocked with 5% milk in Tris-buffered saline (TBS) with 0.1% Tween 20 for 1 h, and incubated with primary anti-pan α GlyR antibody (1:500, rabbit monoclonal IgG; Cat# 146008, Synaptic Systems, RRID:AB_2636914), anti-GlyR β antibody (1:200, rabbit polyclonal IgG; Cat# AGR-014, Alomone, RRID:AB_2340973), and anti-Gβ antibody (1:1000, rabbit polyclonal IgG; Cat# sc-378, Santa Cruz Biotechnology, RRID:AB_631542) overnight at 4°C. After washing steps, the membranes were incubated with anti-rabbit secondary antibodies conjugated to horseradish peroxidase (HRP) (1:5000, goat polyclonal anti-rabbit IgG-HRP, Cat# sc-2004, Santa Cruz Biotechnology, RRID:AB_631746). The immunoreactivity of the proteins was detected using an ECL Plus Western Blotting Detection System (PerkinElmer, Boston, MA, United States). The relative expression of protein was normalized using the expression of the Gβ subunit.

### Behavioral Studies

Male WT and *Glra3*^–/–^ mice of 10–13 weeks old were used in this study, unless otherwise indicated. Mice were allowed to acclimate to the experimental room for at least 1 h prior to behavioral assays. Ethanol was diluted in 0.9% saline (20% v/v) and administered via intraperitoneal (i.p.) injections in doses adjusted by injected volumes.

#### Loss of Righting Reflex

Mice were tested for sedative effects of ethanol using 3.5 g/kg i.p. Mice were injected with ethanol and when mice became ataxic, they were placed in the supine position in V-shaped plastic troughs until they were able to right themselves three times within 30 s. LORR was defined as the time from being placed in the supine position until the righting reflex was regained. It is important to note that the duration of LORR is highly significant because it is the most widely used measure for ethanol sedation/intoxication, in our and other laboratories (Blednov et al., 2012, 2015; Aguayo et al., 2014).

#### Drinking in the Dark

This limited access drinking test produces significant levels of ethanol in the blood (Rhodes et al., 2005). Mice were transferred to individual cages and allowed to acclimate for at least 1 week. Two hours after the lights were turned off, water bottles were replaced with bottles containing 15% v/v of ethanol solution for either 2 h during the first 3 days or 4 h the fourth day. The ethanol bottles were weighed before placement and after removal from the cages every day. In independent experiments, we tested 5% sucrose (w/v) for 2 h (1–3 days) and 4 h (fourth day). The amount of ethanol and sucrose consumed was calculated as g/kg body weight per 2 or 4 h accordingly.

#### Blood Ethanol Concentration

Blood samples from the facial vein from WT and *Glra3*^–/–^ were collected after 10 min on day 4 of drinking in the dark. Blood samples were centrifuged (10,000 rpm × 10 min) and ethanol concentration was determined in the serum using an Analox AM1 Analyzer (Lunenburg, Massachusetts).

### Reagents

Glycine was obtained from Sigma-Aldrich (United States). Ethanol was purchased from Merck Millipore (United States).

### Data Analyses

Results are expressed as the mean ± SE with the following: n.s., not significant; ^∗^*p* < 0.05, ^∗∗^*p* < 0.01, and ^∗∗∗^*p* < 0.001. Statistical analyses were performed using unpaired Student’s t-test or two-way repeated measures ANOVA for studies where each group size was at least *n* = 5. The group size in this study represents independent values. After ANOVA, Bonferroni post hoc test was run only if F achieved the necessary level of statistical significance (*p* < 0.05) and there was no significant variance in homogeneity. As in previous studies (Aguayo et al., 2014; Munoz et al., 2019), in order to obtain statistical power above 95% (α = 0.05, power = 0.95) to determine the existence of statistically significant differences (*p* < 0.05), we used a sample size of 6–8 measurements for the experimental group. For behavioral studies, we considered at least 10–12 animals per group; however, there were some small variations in group size due to unreliable intraperitoneal injections or problems with the bottles. OriginPro 9.0 (Microcal Origin, RRID:SCR_002815, Northampton, MA, United States) software was used for all statistical analyses.

## Results

### Sensitivity to Picrotoxin and Ethanol of α3-Containing GlyRs in HEK293 Cells

**FIGURE 1:**
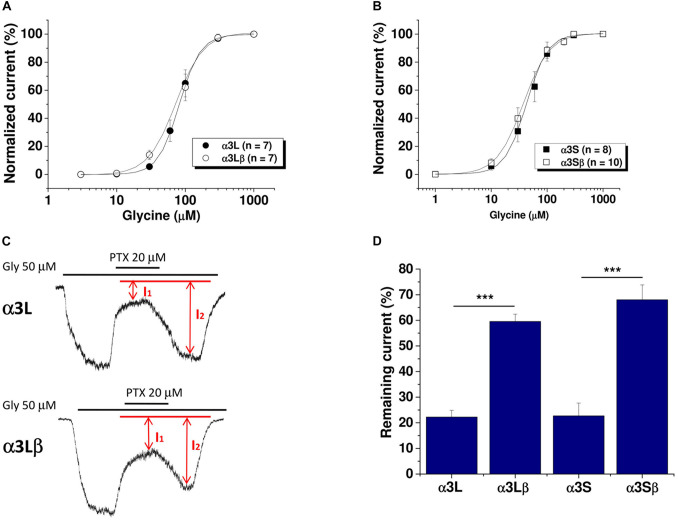
Sensitivity to glycine and picrotoxin of GlyR α3 subunits in HEK293 cells. **(A)** Concentration-response curves for the GlyR α3L variant in homomeric (*n* = 7 for α3L) and heteromeric (*n* = 7 for α3Lβ) receptors. **(B)** Concentration-response curves for the GlyR α3S isoform in homomeric (*n* = 8 for α3S) and heteromeric (*n* = 10 for α3Sβ) receptors. **(C)** Representative traces of glycine-evoked currents for the α3L and α3Lβ variant in the presence of 20 μM picrotoxin. **(D)** The graph shows the current that remained during application of picrotoxin for the different isoforms/combinations of GlyR α3 subunits (*n* = 11 for α3L, *n* = 10 for α3Lβ; *n* = 7 for α3S; and *n* = 5 for α3Sβ). Unpaired Student’s *t*-test. Data are mean ± SEM. ***p < 0.001.

In the mammalian central nervous system, the GlyR α3 subunit is expressed as two splice variants known as α3L and α3S subunits, which differ in that α3L contains a 15-amino acid insertion (TEAFALEKFYRFSDT) within the TM3-TM4 intracellular domain (Nikolic et al., 1998; Eichler et al., 2009). Previous studies from our laboratory showed that homomeric GlyRs containing either α3L or α3S subunits were not modulated by pharmacologically relevant concentrations of ethanol (Sanchez et al., 2015). In the present study, we sought to examine the effect of co-expressing the β subunit in the effect of ethanol potentiation on GlyR α3 subunits using HEK293 cells. The results in Figures 1A,B show that the co-expression of β and α subunits did not alter the EC_50_ value of the concentration-response curve of α3L (α3L: 82 ± 7 μM versus α3Lβ: 72 ± 9 μM; Figure 1A) or α3S (α3S: 43 ± 4 μM versus α3Sβ: 37 ± 5 μM; Figure 1B).

Previous studies have reported that PTX is able to differentially block homomeric and heteromeric combinations of GlyRs (Pribilla et al., 1992). It was shown that heteromeric αβ GlyRs exhibits a reduced sensitivity to PTX, while homomeric GlyRs are more sensitive to PTX blockade (Maleeva et al., 2017). In order to determine the sensitivity of the different combinations of GlyR α3 and β subunits to PTX, we used an EC_30_ of glycine from the recorded concentration-response curve and quantified the percentage of current that remained after application of 20 μM PTX (Figure 1C). The results obtained revealed that the presence of β subunits conferred resistance to the inhibitory effect of PTX to the GlyR. For example, the values of remaining current after PTX application were 22 ± 3% for α3L and 59 ± 3% for α3L; and 23 ± 5% for α3S and 68 ± 6% for α3Sβ (^∗∗∗^*p* < 0.001; Figure 1D). Thus, the data is in agreement with that previously reported (Pribilla et al., 1992). Indeed, co-expression of β together with α3 subunits confers PTX resistance to the GlyR and supports the presence of a heteropentameric receptor.

**FIGURE 2:**
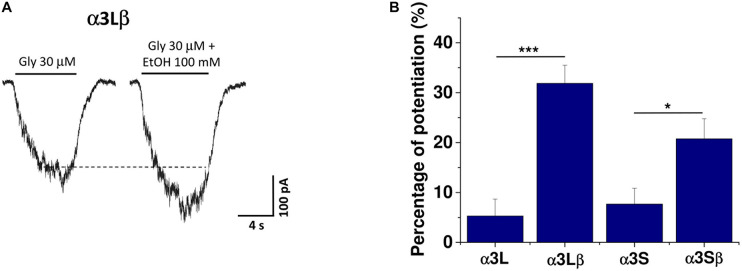
Ethanol sensitivity of different GlyR α3 subunits in HEK293 cells. **(A)** Representative current traces using an EC_10_ glycine in the absence and presence of 100 mM ethanol of α3Lβ subunits. **(B)** The graph shows the quantification of ethanol potentiation for the different combinations of GlyR α3 subunits in HEK293 cells (*n* = 10 for α3L, *n* = 11 for α3Lβ; *n* = 6 for α3S; and *n* = 11 for α3Sβ). The presence of the β subunit confers ethanol sensitivity to the α3 subunit. Unpaired Student’s *t*-test. Data are mean ± SEM. ***p < 0.001; *p < 0.05.

Using a concentration corresponding to an EC_10_ from the concentration-response curve in Figure 1, we evaluated the sensitivity of the different α3 subunit isoforms, either alone or in combination with β subunits, to 100 mM ethanol. As previously demonstrated (Sanchez et al., 2015), homomeric α3L and α3S subunits were not significantly potentiated by ethanol (α3L: 5 ± 3% and α3S: 7 ± 3%; Figure 2B). Interestingly, when the β subunit was co-expressed with the α3L isoform, we detected a significant increase in the ethanol potentiation (α3Lβ: 32 ± 4% versus α3L: 5 ± 3%; Figure 2B; ^∗∗∗^*p* < 0.001). Figure 2A shows representative current traces in the absence and presence of ethanol in the heteromeric α3Lβ GlyR. Similar results were found using the α3S isoform, although to a lesser extent when compared to α3L (α3Sβ: 21 ± 4% versus α3S: 7 ± 3%; Figure 2B; ^∗^*p* < 0.05).

### Electrophysiological Properties of Dissociated Neurons From Nucleus Accumbens in *Glra3*^–/–^ Mice

**FIGURE 3:**
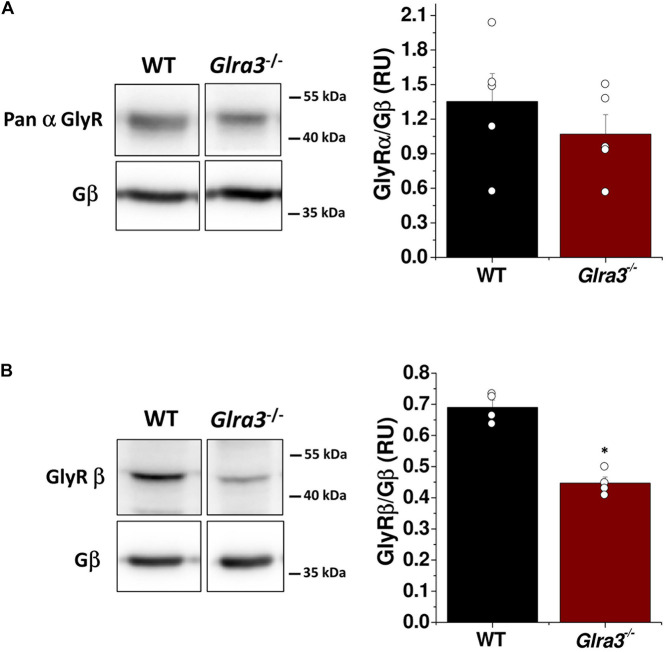
Presence of GlyR α and β subunits in the nAc of WT and *Glra3*^–/–^ mice. **(A)** Western blot of lysates from the nAc of WT and *Glra3*^–/–^ mice for GlyR α and β subunits. The graph shows similar levels of α subunits in *Glra3*^–/–^ mice compared to WT mice (*n* = 5 WT and *n* = 5 *Glra3*^–/–^). **(B)** Western blot and graph show low levels of the β subunit in the *Glra3*^–/–^ mice compared to WT mice (*n* = 4 WT and *n* = 4 *Glra3*^–/–^). Unpaired Student’s *t*-test. Data represent mean ± SEM. ^∗^*p* < 0.05.

Previous studies have shown that mouse accumbal neurons express both GlyR α and β subunit mRNAs (Jonsson et al., 2012; Munoz et al., 2019). In order to examine protein expression levels, we performed Western blot experiments in tissue lysates from the nAc dissected from brains slices of WT and *Glra3*^–/–^ mice using pan α and β subunit antibodies. A comparison of protein levels, reflected by the densitometry analysis, for the α subunit in WT and *Glra3*^–/–^ mice indicates that α3 represents a small component of total GlyR α subunits, since the total α subunit was only slightly reduced in *Glra3*^–/–^ mice (ns; Figure 3A). The presence of the β subunit, a protein important for the anchoring of GlyRs to synaptic sites (Grudzinska et al., 2005), was also analyzed in the *Glra3^–/–^* mice using Western blot. Remarkably, the results revealed a significant reduction in the expression of GlyR β subunits in *Glra3^–/–^* mice when compared to WT mice (^∗^*p* < 0.05; Figure 3B).

**FIGURE 4:**
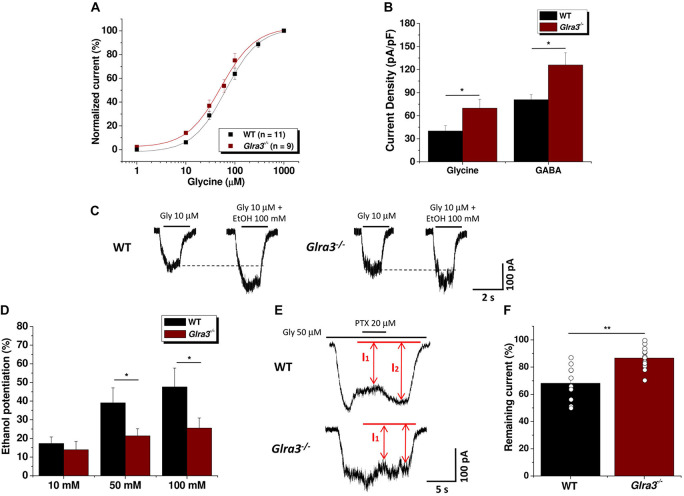
Electrophysiological properties of dissociated neurons from the nAc in wild-type and *Glra3*^–/–^ mice. **(A)** The graph shows the glycine concentration-response curves in accumbal neurons from WT (*n* = 11; black squares) and Glra3^–/–^ (*n* = 9; red squares) mice. **(B)** The graph shows the current density of dissociated accumbal neurons from WT and *Glra3*^–/–^ mice for glycine (*n* = 21 for WT and *n* = 27 for *Glra3*^–/–^) and GABA (*n* = 11 for WT and *n* = 19 for *Glra3*^–/–^). **(C)** Representative traces of glycine-evoked currents (EC_10_) in the absence and presence of 100 mM ethanol in accumbal neurons from WT and *Glra3*^–/–^ mice. **(D)** The graph summarizes the effect of ethanol concentrations in the nAc from *Glra3*^–/–^ and WT mice (*n* = 8 for WT and *n* = 15 for *Glra3*^–/–^). **(E)** Representative traces of glycine-evoked current from accumbal neurons from WT and *Glra3*^–/–^ mice in presence of 20 μM picrotoxin. **(F)** The graph summarizes the percentage of the current that remained during application of picrotoxin in the WT and *Glra3*^–/–^ mice (*n* = 11 for WT, *n* = 14 for *Glra3^–/–^* mice). Unpaired Student’s *t*-test. Data are mean SEM. **p < 0.01; *p < 0.05.

Previous studies from our laboratory have demonstrated the presence of functional ethanol-sensitive GlyRs in the nAc (Forstera et al., 2017) with a predominance of α1 and α2 subunits (Jonsson et al., 2012; Forstera et al., 2017). However, a recent study showed that accumbal neurons did not express any noticeable tonic current in *Glra3*^–/–^ mice, suggesting that α3-containing GlyRs were fundamental in this region (McCracken et al., 2017). In order to study the contribution of α3 subunits to general electrophysiological properties, we examined dissociated accumbal neurons in WT and *Glra3*^–/–^ mice (up to 8 weeks old) and found no significant differences in the concentration-response curves (EC_50_ of 51 ± 9 μM for *Glra3*^–/–^ mice, and EC_50_ of 65 ± 8 μM for WT; Figure 4A). However, we found significant differences in current density parameters in response to supramaximal concentrations of glycine and GABA (Figure 4B). Analysis of current density in response to glycine showed that *Glra3*^–/–^ mice had higher values (69 ± 11 pF/pA) compared to WT mice (40 ± 7 pF/pA) (^∗^*p* < 0.05; Figure 4B). Similarly, the current density of the GABA_A_ mediated response also revealed higher values in the *Glra3*^–/–^ mice (126 ± 16 pF/pA) compared to WT mice (81 ± 7 pF/pA) (^∗^*p* < 0.05; Figure 4B). In order to evaluate the effect of the presence of α3-containing GlyRs in the nAc on the potentiation by ethanol, we performed electrophysiological recordings using a glycine concentration corresponding to an EC_10_ in the presence of different ethanol concentrations in accumbal neurons from *Glra3*^–/–^ and WT mice. This analysis revealed a significant reduction in potentiation using 50 and 100 mM of ethanol in *Glra3*^–/–^ mice (Figure 4C). These results demonstrated that ethanol potentiated the current in WT mice by 17 ± 3% at 10 mM, 39 ± 8% at 50 mM, and 48 ± 10 at 100 mM; whereas in *Glra3*^–/–^ mice the ethanol potentiation decreased to 14 ± 4% at 10 mM, (21 ± 4% at 50 mM, and 25 ± 5% at 100 mM (^∗^*p* < 0.05, Figure 4D). These results suggest that α3 subunits, like α1 and α2, play a role in ethanol sensitivity of GlyRs present in the nAc.

Since it has been previously reported that homomeric and heteromeric GlyRs have differential sensitivity to picrotoxin (Pribilla et al., 1992; Maleeva et al., 2017), we performed electrophysiological recordings in accumbal dissociated neurons using picrotoxin to determine the possible subunit arrangement of GlyR α3 and β subunits in the nAc. The results showed that application of 20 μM picrotoxin to accumbal neurons only slightly affected the amplitude of the glycine-induced current, indicating the presence of a heteromeric receptor. We found that the current in *Glra3*^–/–^ mice was less affected by PTX (68 ± 4% of control for WT; 87 ± 2% of control for *Glra3*^–/–^ mice) (^∗∗^*p* < 0.01) (Figures 4E,F). The smaller effect of PTX found in the *Glra3*^–/–^ mice suggests an increase in αβ complexes in the membrane supporting a compensatory effect by different GlyR subtypes.

### Ethanol-Induced Sedation Is Not Altered in Male *Glra3*^–/–^ Mice

The genetically modified *Glra3*^–/–^ mice did not display visible abnormalities or foot clasping behavior when the mouse was lifted by the tail (Supplementary Figure 1A) indicating the absence of alterations in muscle tone and coordination as seen in *Glra1* mutant mice (Schaefer et al., 2018) and *Glra2*^–/–^ mice (San Martin et al., 2020). Additionally, this knockout mouse did not exhibit gross alterations in muscle strength when assayed by a qualitative grip test (Supplementary Figure 1B). This is in line with previous studies using the same *Glra3*^–/–^ mouse model (Harvey et al., 2004; Blednov et al., 2015).

**FIGURE 5:**
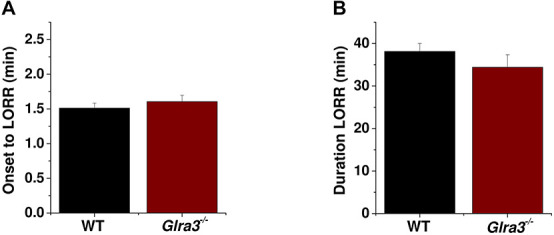
Effect of high doses of ethanol in male *Glra3*^–/–^ mice. **(A)** The loss of righting reflex (LORR) assay was used as an index of central nervous system depression. Mice received an intraperitoneal injection of 3.5 g/kg of ethanol and the latency to LORR was measured. *Glra3*^–/–^ mice did not differ in their reflex ability compared to WT mice. **(B)** The ability to recover the righting reflex was measured as duration of LORR, where *Glra3*^–/–^ mice recovered at similar times compared to WT mice (*n* = 10 for WT and *n* = 9 for *Glra3*^–/–^). Unpaired Student’s *t*-test. Data are mean ± SEM.

To study the contribution of GlyR α3 subunits to the effect of intoxicating doses of alcohol, we performed the loss of righting reflex (LORR) assay in *Glra3*^–/–^ and WT mice. After an injection of 3.5 g/kg of ethanol i.p., we did not find differences in the onset to LORR between male WT and *Glra3*^–/–^ mice (1.5 ± 0.1 min for WT; 1.6 ± 0.1 for *Glra3^–/–^*) (ns; Figure 5A) or in the duration of LORR (38 ± 2 min for WT; 34 ± 3 min for *Glra3*^–/–^) (ns; Figure 5B).

### Male *Glra3*^–/–^ Mice Had Increased Ethanol Consumption Compared to Wild-Type Mice

**FIGURE 6:**
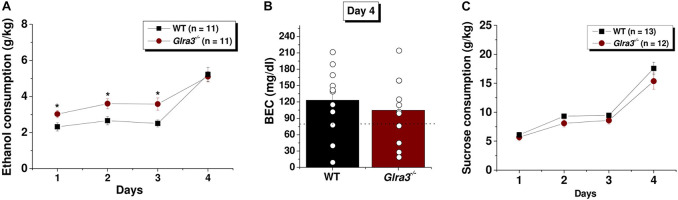
Male *Glra3*^–/–^ mice consume higher amounts of ethanol in the drinking in the dark test. **(A)** Graph summarizes the drinking in the dark (DID) test in male WT and *Glra3*^–/–^ mice. During the dark phase of the day, the mice were able to drink a 15% v/v ethanol solution for 2 h the first 3 days. On the fourth day, mice were allowed to drink for 4 h. Male *Glra3*^–/–^ mice consumed more ethanol on day 1, 2, and 3 compared to WT mice (measured in grams of ethanol per kilogram of weight (g/kg); (*n* = 11 for WT and *n* = 11 for *Glra3*^–/–^). Two-way repeated measures ANOVA and Bonferroni *post hoc* test. **(B)** The graph shows that after the 4th day of consumption, *Glra3*^–/–^ mice had similar blood ethanol concentrations (BEC) as WT mice. **(C)** The graph shows that sucrose consumption was unaltered in *Glra3*^–/–^ compared to WT mice (*n* = 13 for WT and *n* = 12 for *Glra3*^–/–^). Unpaired Student’s *t*-test. Data are mean ± SEM. *p < 0.05.

An important number of studies have demonstrated that GlyRs present in the reward system are relevant for ethanol consumption and preference (Molander and Soderpalm, 2005a; Lido et al., 2011; Li et al., 2012; Munoz et al., 2019). Interestingly, the increase in dopamine levels in the nAc in response to ethanol is affected by GlyR activation (Molander and Soderpalm, 2005b). The drinking in the dark (DID) assay has been previously described as a paradigm to evaluate behaviors associated with ethanol consumption (Rhodes et al., 2005). Our results showed that *Glra3*^–/–^ mice had significantly higher ethanol consumption compared to WT mice during the 2-h consumption phase of the test (Day 1: 2.3 ± 0.2 g/kg in WT versus 3.0 ± 0.2 in *Glra3*^–/–;^ Day 2: 2.6 ± 0.2 g/kg in WT versus 3.6 ± 0.3 in *Glra3*^–/–^; Day 3: 2.5 ± 0.2 g/kg in WT versus 3.5 ± 0.3 in *Glra3*^–/–^, ^∗^*p* < 0.05; Figure 6A). In agreement with the drinking data on day 4, analysis of blood ethanol concentration (BEC) showed that *Glra3*^–/–^ mice had similar blood levels of ethanol compared to WT mice after 4 h of consumption (123 ± 19 mg/dl for WT versus 105 ± 18 mg/dl for *Glra3*^–/–^, ns; Figure 6B). Furthermore, no significant differences were found in sucrose consumption between male *Glra3*^–/–^ and WT mice (ns; Figure 6C).

## Discussion

GlyRs are neurotransmitter-gated ion channels of the Cys-loop receptor family and mediate fast inhibitory neurotransmission in the nervous system and retina (Lynch, 2009). While GlyR α1 and α2 subunits form functional Cl^–^ channels that are widely expressed in spinal cord and brainstem neurons, GlyRs containing α3 subunits are thought to be expressed more restrictively in neurons associated to inflammatory pain sensitization and rhythmic breathing (Harvey et al., 2004; Manzke et al., 2010). It is also now accepted that α3-containing GlyRs are also expressed in other brain regions (Eichler et al., 2009; Delaney et al., 2010; Aroeira et al., 2011; Jonsson et al., 2012), and more recent studies using electrophysiological recordings in brain slices and animal behavior suggested the presence of α3-containing GlyRs in basal ganglia (Blednov et al., 2015; McCracken et al., 2017). In the present study, we tested the influence of the β subunit on the ethanol sensitivity of GlyR α3 subunits in HEK293 cells and accumbal neurons. Although our data indicate that the KO mice display compensations in GlyR and GABA_A_R, it appears that the heteromeric α3β complex might be linked to elevated alcohol drinking.

### Sensitivity of GlyR α3 Subunits to Ethanol and Picrotoxin

It is broadly accepted that GlyRs expressed in several brain regions are sensitive to pharmacological concentrations of ethanol (Aguayo et al., 1996; Ye et al., 2001; Forstera et al., 2017). In many studies, α1 and α2 are the subunits reported to be more responsible for these effects (Aguayo et al., 2014; Gallegos et al., 2021). Previously, α3 subunits were not considered to be a sensitive target for ethanol modulation because it was reported that they were not sensitive to ethanol when expressed as homomeric GlyRs (Sanchez et al., 2015). In the present study, we found that when α3L and α3S subunits were co-expressed with β GlyRs, they became more sensitive to ethanol. Thus, the α3 subunit appears to be important in the mesolimbic system. In addition, it was reported in adult hippocampus and was up-regulated in temporal lobe epilepsy (Eichler et al., 2009). The present results show that the presence of the β subunit does not modify receptor properties like EC_50_ values for either the α3S or α3L variant. It was previously reported that the presence of β subunits in GlyRs diminished the blocking action of PTX (Pribilla et al., 1992; Maleeva et al., 2017). In agreement, using the same methodology used by Maleeva et al. (2017), we found that the α3L and α3S variants co-expressed with the β subunit showed decreased PTX sensitivity than α3S or α3L subunits alone.

### Role of GlyR α3 Subunits in Nucleus Accumbens Neurons

Previous studies demonstrated the expression of GlyR α subunits in the nAc (Jonsson et al., 2012), an important basal ganglia region that integrates information from cortical and limbic structures and initiates goal-directed behaviors (Scofield et al., 2016). In order to study the contribution of GlyR α3 subunits in accumbal neurons to electrophysiological properties and alcohol-related behaviors, we used *Glra3*^–/–^ mice (Harvey et al., 2004). While Western blot analysis showed no significant changes in global α subunit levels in the nAc in *Glra3*^–/–^ mice, the results showed a significant decrease in GlyR β subunits in the knockout mice. These results indicate that deletion of *Glra3* only slightly reduced the presence of the α subunit pool in the nAc, thus suggesting the presence of a lower level of α3 as compared to α1 and α2 (Forstera et al., 2017; San Martin et al., 2020; Gallegos et al., 2021). However, the data does not allow us to rule out the possibility that compensatory up-regulation of α subunits occurred in the *Glra3*^–/–^ mice. In fact, the significant reduction in the overall β subunit levels at first suggested compensation by homomeric α1 and/or α2 subunit GlyRs. However, functional data show that PTX inhibited the current to a smaller extent in *Glra3*^–/–^ mouse. When compared to the *Glra2*^–/–^ mice (San Martin et al., 2020), the *Glra3*^–/–^ mouse showed a much higher glycine current density in dissociated accumbal neurons. Thus, the α3 subunit appears to control brain development in a divergent manner to α2 (Avila et al., 2013).

A recent study suggested a critical role of α3 subunits in the glycinergic current in brain slices of nAc using the *Glra3*^–/–^ mice (McCracken et al., 2017). Surprisingly, in *Glra3*^–/–^ mice we found increases in glycine- and GABA-activated current densities, suggesting higher levels of functional receptors in response to the gene knockout. The more significant difference in methodology with the previous study was that we used dissociated accumbal neurons to determine in a direct approach their sensitivity to glycine and ethanol. We found that accumbal neurons from *Glra3*^–/–^ mice were less sensitive to several concentrations of ethanol. Although the increase in current density does not correlate with the total level of protein of α and β subunits detected, we believe that the most significant differences are at the level of functional proteins (ion channel formation) and sensitivity to ethanol. In terms of functional proteins, we suggest that an important extent of GlyRs might undergo endocytosis in the WT mice, while in the *Glra3*^–/–^ all GlyRs are expressed in the membrane and are functional, leading to an increase in current density. The mechanism for this change is not resolved and future studies should expand on whether this effect reflects a lower content in α3 subunits or a compensatory alteration in other subunits.

### Behavioral Effect of Ethanol in the *Glra3*^–/–^ Mouse

Acute consumption of intoxicating doses of ethanol produces rapid changes in brain functions varying from lack of coordination, motor ataxia and sedation, to respiratory depression, coma, and death at higher doses of ethanol. On the other hand, chronic use is associated with alcohol seeking behavior, binge drinking, tolerance, and dependence (Spanagel, 2009). Previous studies implicated the activation of GlyRs in the modulation of dopamine release in the nAc (Molander and Soderpalm, 2005a,b; Lido et al., 2011) and subsequently in ethanol consumption (Molander et al., 2005). A previous study of alcohol-related behaviors in *Glra3*^–/–^ mice (Blednov et al., 2015) showed the presence of an enhanced drinking level. However, the study did not examine the expression of the GlyR subunits and functional significance in regions important for addiction. The present results showed that intoxicating doses of ethanol did not alter the time of onset and duration of LORR in *Glra3*^–/–^ mice compared to WT controls, revealing that α3 subunits do not play a critical role in regulating awakening, sedation, and loss of consciousness. This unchanged duration of LORR in *Glra3*^–/–^ mice is consistent with previous published findings (Blednov et al., 2015) and might support a more restricted localization of α3 subunits in the CNS unable to produce a sedative action. On the other hand, previous studies have demonstrated that α1 and α2 subunits are important for the sedative effects of ethanol (Aguayo et al., 2014; San Martin et al., 2020; Gallegos et al., 2021). Thus, these findings suggest that α1 and α2 subunits are more relevant than α3 subunits in terms of alcohol effects on GlyRs in the CNS.

Repeated cycles of binge drinking and abstinence are key components in the development of dependence (Gowin et al., 2017). We used a DID paradigm to assess whether there is a difference in the consumption pattern between *Glra3*^–/–^ mice and controls. An important feature of this assay is that mice self-administer alcohol and reach blood ethanol concentrations (BEC) that mirror levels of binge-like drinking behavior (Rhodes et al., 2005). The National Institute of Alcohol Abuse and Alcoholism define binge drinking as a form of abusive alcohol drinking to obtain a blood ethanol concentration of at least 80 mg/dl, which typically occurs after 4 drinks for women and 5 drinks for men, in about 2 h (Patrick and Azar, 2018). Our results showed a significant higher consumption of ethanol on day 1, 2, and 3 in *Glra3*^–/–^ mice when compared to WT controls. The pattern of consumption is similar to that observed in a *Glra2* knockout mouse (San Martin et al., 2020), in which the ethanol intake was immediate when exposed to ethanol and not a gradual consumption like WT animals. Although it has been shown that the α3 subunit is present in primary somatosensory neurons (Bae et al., 2016), the differences in ethanol consumption are not attributable to alteration in taste, since we found that sucrose intake was similar in the two genotypes. The ethanol intake results agree with a study by Blednov et al. (2015) that showed that *Glra3*^–/–^ mice increased ethanol intake and preferences in the 24-h intermittent access assay. Overall, the present results agree with previous studies that showed that GlyRs are critical ethanol targets that control alcohol intake, for example, mice with ethanol-insensitive α1 and α2 subunit GlyRs (KI mice) (Munoz et al., 2019; Gallegos et al., 2021) have increased alcohol drinking. Similarly, the loss in α2-containing GlyRs (*Glra2* knockout mouse) results in mice having a high ethanol consumption (San Martin et al., 2020).

In summary, the present study using a mouse lacking the GlyR α3 subunit supports the existence of low, but significant levels of α3 subunits in the nAc. The differences found in ethanol sensitivity and consumption suggest that GlyRs are a biologically relevant target for the regulation of the reward system and the rewarding properties of alcohol. Future studies will determine if neuronal compensations in the KO mice have an effect on the present interpretation that the α3 GlyR subunit may have in ethanol drinking behavior.

## Data Availability Statement

The original contributions presented in the study are included in the article/Supplementary Material, further inquiries can be directed to the corresponding author.

## Ethics Statement

The animal study was reviewed and approved by the University of Concepción and conducted according to the ethical protocols established by the National Institutes of Health (NIH, United States).

## Author Contributions

LS and LA participated in research design. RH designed and generated *Glra3* KO mice. LS, LA-W, and AA performed the experiments and analyzed the data. LS, LA, GY, and RH wrote or contributed to the writing of the manuscript. All authors reviewed the manuscript.

## Conflict of Interest

The authors declare that the research was conducted in the absence of any commercial or financial relationships that could be construed as a potential conflict of interest.

## Publisher’s Note

All claims expressed in this article are solely those of the authors and do not necessarily represent those of their affiliated organizations, or those of the publisher, the editors and the reviewers. Any product that may be evaluated in this article, or claim that may be made by its manufacturer, is not guaranteed or endorsed by the publisher.

## Abbreviations

AUD: alcohol use disorder
DID: drinking in the dark
GlyR: glycine receptor
nAc: nucleus accumbens
LORR: loss of righting reflex
PFC: prefrontal cortex
STN: strychnine
VTA: ventral tegmental area.
