# Supplementary material for: Contribution of GlyR α3 Subunits to the Sensitivity and Effect of Ethanol in the Nucleus Accumbens
Source: Front Mol Neurosci. 2021 Oct 22;14:756607. doi: 10.3389/fnmol.2021.756607 (PMC8570041; doi:10.3389/fnmol.2021.756607)
Supplement: Supplementary file 1 [file Image_1.PDF]

# Supplementary Figure S1

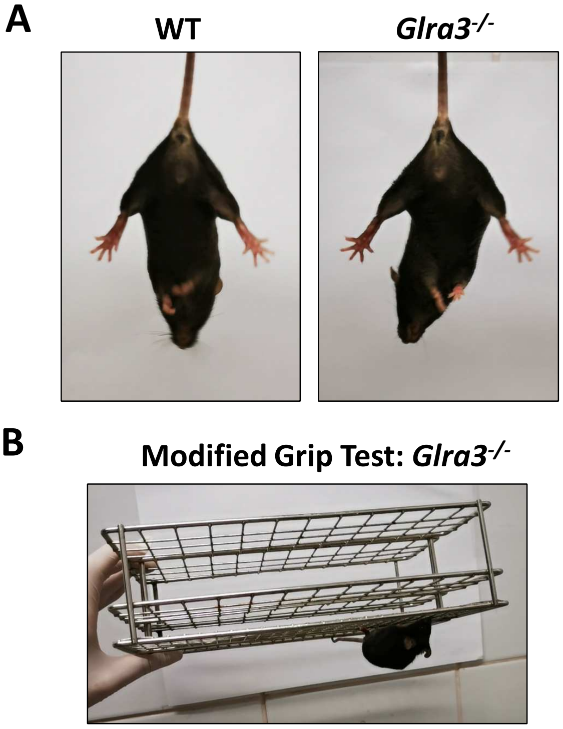


**Supplementary Figure S1. *Glra3*$^{-/-}$ mice did not show gross behavioral or morphological phenotypes.** (A) The *Glra3*$^{-/-}$ mice did not display an increase in muscle tone as reflected by lack of limb-clenching behavior. (B) Modified grip test showed no alteration in muscle strength in *Glra3*$^{-/-}$ mice.
